# Current insights in ICU nutrition: tailored nutrition

**DOI:** 10.1097/MCC.0000000000001016

**Published:** 2023-01-27

**Authors:** Anoek Jacqueline Hubertine Hermans, Babette Irene Laarhuis, Imre Willemijn Kehinde Kouw, Arthur Raymond Hubert van Zanten

**Affiliations:** a Department of Intensive Care Medicine, Gelderse Vallei Hospital, Ede; b Wageningen University & Research, Division of Human Nutrition and Health, Wageningen, The Netherlands

**Keywords:** body impedance analysis, energy, intensive care, proteins, timing

## Abstract

To summarize recent research on critical care nutrition focusing on the optimal composition, timing, and monitoring of enteral feeding strategies for (post)-ICU patients. We provide new insights on energy and protein recommendations, feeding intolerance, and describe nutritional practices for coronavirus disease 2019 ICU patients.

**Recent findings:**

The use of indirect calorimetry to establish individual energy requirements for ICU patients is considered the gold standard. The limited research on optimal feeding targets in the early phase of critical illness suggests avoiding overfeeding. Protein provision based upon the absolute lean body mass is rational. Therefore, body composition measurements should be considered. Body impedance analysis and muscle ultrasound seem reliable, affordable, and accessible methods to assess body composition at the bedside. There is inadequate evidence to change our practice of continuous enteral feeding into intermittent feeding. Finally, severe acute respiratory syndrome coronavirus 2 patients are prone to underfeeding due to hypermetabolism and should be closely monitored.

**Summary:**

Nutritional therapy should be adapted to the patient's characteristics, diagnosis, and state of metabolism during ICU stay and convalescence. A personalized nutrition plan may prevent harmful over- or underfeeding and attenuate muscle loss. Despite novel insights, more research is warranted into tailored nutrition strategies during critical illness and convalescence.

## INTRODUCTION

Critical care nutrition is a rapidly evolving field in which significant steps have been made toward nutritional recommendations specific to each patient. This shift allows healthcare providers to consider the patient's characteristics, medical diagnosis, current treatments, and metabolic state [1]. The multifactorial nature of nutritional needs in critically ill patients and the difference in outcomes and methodologies assessed in available studies pose challenges to establishing fitting guidelines.

This narrative review aims to summarize the latest updates on nutritional practices in the ICU. It focuses on energy content, protein provision, mode of enteral feeding, and timing of enteral nutrition. Also, the latest insights into nutritional strategies for severe acute respiratory syndrome coronavirus 2 (SARS-CoV-2)-infected ICU patients are addressed. Finally, as enteral feeding intolerance (FI) is associated with worse outcomes, such as higher mortality and fewer ventilator-free days [2], clinical implications and treatment of FI according to the latest nutritional recommendations are evaluated. 

**Box 1:**
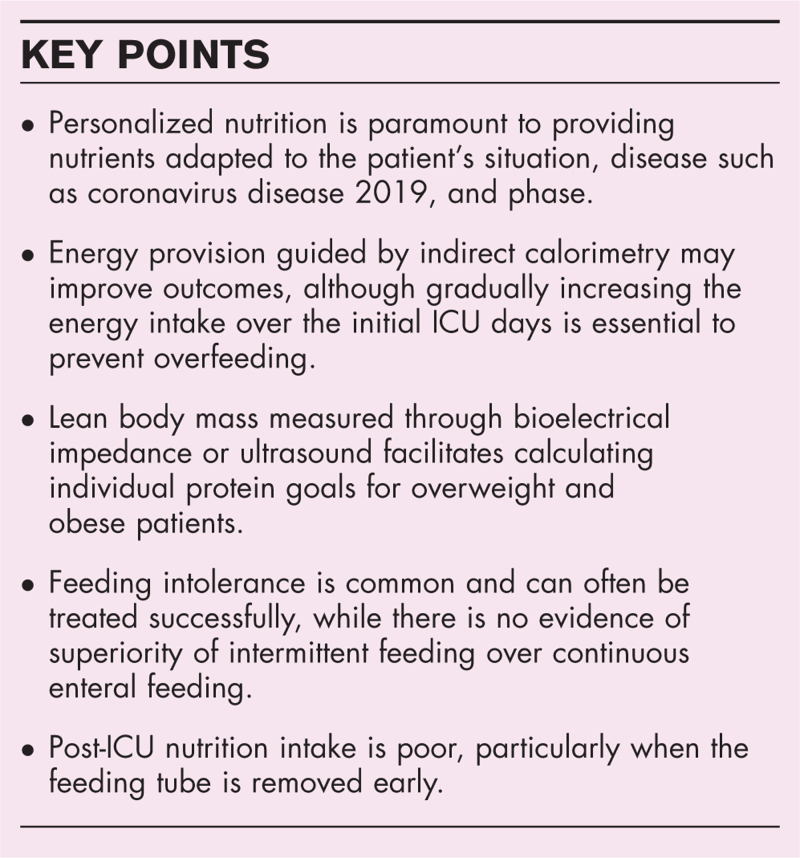
no caption available

## COMPOSITION OF ENTERAL NUTRITION

Nutrition provision, matching an individual's needs, is crucial to enhance recovery and decrease complications in critical illness. The appropriate composition of enteral nutrition (EN) includes adequate amounts of energy, specific macronutrient composition, and the addition of essential micronutrients.

## PROTEIN PROVISION

Catabolism in critical illness is known to encompass proteolysis of body proteins, resulting in rapid loss of muscle mass. Muscle wasting leads to muscle weakness and impaired metabolic health. Decreased functional performance has been observed in ICU survivors up to multiple years post-ICU [3].

Muscle mass maintenance is regulated through muscle protein synthesis and breakdown rates, with periods of muscle protein anabolism being key to maintaining muscle mass. Dietary protein is an anabolic stimulus for muscle protein synthesis in healthy subjects. Therefore, augmented protein intake has been suggested as an effective strategy to attenuate muscle wasting. Observational studies have shown higher protein delivery to improve clinical outcomes, for example, reduced mortality. Therefore, international guidelines recommend a protein provision of 1.2–2.0 g/kg/day [4,5]. However, these guidelines have been based on retrospective and prospective cohort studies, lacking data on the effect of protein provision on functional and metabolic outcomes.

A recent retrospective study by Lambell *et al.* analysed protein provision and muscle mass loss (assessed by using CT-derived skeletal muscle area). Although skeletal muscle area declined over the first three weeks of ICU stay, with protein provision averaging 1.1 g/kg/day (and 83% being achieved), protein delivery was not associated with muscle loss [6]. Another retrospective multicentre database study (*N* = 21 100) that compared a standard (0.8–1.2 g/kg/day) vs. low protein diet (<0.8 g/kg/day) showed a lower hospital mortality in patients that received a late standard protein diet (0.8–1.2 g/kg/day) vs. patients that received a continuously low protein diet (<0.8 g/kg/day). This benefit was not further exaggerated in the group that received a late high protein diet (>1.2 g/kg/day) [7].

Findings align with a recent meta-analysis of 19 RCTs that compared higher vs. lower protein delivery (with matched energy delivery between groups) on clinical and patient-centred outcomes showing no further improvement in physical function and mortality in response to high-protein diets. It must be noted that the nutritional goals in the included RCTs were often not met, with intake ranges varying from 0.9 to 2.6 g/kg/day. The relatively low protein intake in these ‘high-protein’ groups complicates the comparison to retrospective data. Moreover, some studies delivered total protein goals on the first ICU day, whereas others gradually increased protein provision in the early phase.

However, in five studies, associations between protein provision and muscle loss suggested that higher protein delivery attenuated skeletal muscle loss by ∼3.4% per week [8^▪▪^]. In health, muscle mass declined under high protein provision without resistance training and increased when high resistance training was added to the regimen [9]. Therefore, early resistance training might preserve muscle mass and mitigate muscle loss during critical illness.

Quantifying amino acid balance during ICU admission and assessing whole-body and muscle protein metabolism in response to increased protein intakes provides mechanistic insight into the muscle protein anabolic capacity in ICU patients [10^▪^]. Contemporary stable isotope methodology assessing whole-body and muscle protein anabolic response to protein delivery has only been addressed by a few studies [11], as this technique is expensive, labour-intensive, and requires repeated blood sampling and skeletal muscle tissue collection [12]. Chapple *et al.*[10^▪^] quantified postprandial protein handling in response to duodenal protein feeding in ICU patients and BMI and age-matched healthy control subjects. Although dietary amino acid uptake was similar between groups, the deposition of dietary amino acids into myofibrillar protein was 60% lower after the protein bolus. This study demonstrates profound skeletal muscle anabolic resistance in critical illness, likely contributing to muscle wasting. Whether higher protein provision can overcome this critical illness anabolic resistance warrants further studies.

More studies have been conducted to assess protein needs with the increasing availability of bedside body composition measurements. International guidelines have recently recommended assessing lean body mass (LBM) to determine protein goals in obese and overweight patients [4]. Several predictive formulas provide estimations of LBM) and can differ significantly from actual LBM [13^▪^]. LBM or muscle mass can be better estimated using Dual-energy X-ray absorptiometry, CT or MRI scans. However, these methods are impractical or impossible to implement at the bedside, are costly and cannot be repeated regularly [14].

Bioelectric impedance analysis (BIA) is a more affordable and practical method to assess LBM in ICU patients. While fluid overload can introduce variations in measurements [14], multifrequency BIA can assess the extracellular water surplus, which can be adjusted to prevent LBM overestimation [15]. Bedside ultrasonography is an alternative to assess LBM but more operator-dependent.

High protein provision (i.e., >1.2 g/kg/day) has not yet been proven to improve clinical endpoints compared to lower intake levels in ICU patients. However, it may attenuate muscle loss. Assessment of LBM by BIA is recommended, especially in obese and overweight patients. Areas that remain to be investigated are combining high protein provision with early resistance therapy, reliable and affordable methods to assess muscle anabolism, and practical methods to assess the whole-body protein balance.

## ENERGY INTAKE AND ENERGY EXPENDITURE

Determining energy requirements is essential to prevent harmful under- and overfeeding. However, the optimal amount of energy provision remains debatable. Energy expenditure (EE) may vary during different phases of critical illness. The nutritional status and endogenous energy production account for significant proportions of energy substrate during early critical illness. The resting energy expenditure (REE) can be measured using indirect calorimetry (IC). As ICU patients typically engage in minimal physical activity, REE will be close to the total energy expenditure (TEE). Predictive formulas differ significantly from indirect calorimetry REE and can lead to deviations up to 1000 kcal/day from the actual EE [16]. Duan *et al.*[17^▪▪^] showed that IC-guided energy delivery reduces short-term mortality by 23%, probably by preventing harmful under- or overfeeding. However, the recent TICACOS-II trial could not reproduce this mortality effect, although underpowering may have played a role [18].

IC does not account for endogenous energy production, noninhibitable by exogenous feeding or insulin, typically present in the early phase. No reliable bedside method to assess this production has been established yet. Therefore, indirect calorimetry remains the preferred method to assess energy needs after the initial phase of high endogenous energy production has resolved [19,20]. If indirect calorimetry is unavailable, *V*CO_2_ measurements (kcal/24 h = *V*CO_2_ × 8.19) are slightly more accurate than predictive formulas [4]. However, in a study from our group, *V*CO_2_ overestimates the actual EE compared to indirect calorimetry [21^▪^].

## TIMING AND NUTRITION TARGETS

Besides the macronutrient composition of EN, the timing of nutritional provision is important as energy and protein needs vary over stages of critical illness.

## PREVENTION OF OVERFEEDING IN THE EARLY PHASE

During the early phase of critical illness, endogenous energy production is estimated to be 500–1400 kcal/day. Therefore, guidelines recommend gradually increasing energy intake over several days up to 80–100% of the REE to prevent overfeeding. In observational studies, a hypocaloric intake of 70–80% of the REE in the early phase of critical illness was associated with reduced mortality [4]. Conversely, the majority of RCTs did not confirm these observations. A recent systematic review by Zhou *et al.*[22^▪^] observed no effect of hypocaloric feeding in the early phase of critical illness, with matched protein intake levels, on mortality and ICU or hospital length of stay. However, this systematic review included studies using predictive formulas and IC-derived measurements to calculate EE.

Future studies using only IC-derived EE are needed to elucidate the need and exact timing of (hypocaloric) feeding in early critical illness.

## FEEDING MODALITIES

The mode of enteral feeding has been debated for years. Commonly used enteral feeding modalities include continuous (24 h/day), intermittent, bolus, or cyclic feeding [23]. Although international guidelines recommend continuous feeding, this recommendation is based on limited evidence [4].

A recent RCT by Lee *et al.*[24^▪^] demonstrated improved feeding adequacy among patients with continuous feeding; >80% of the nutritional target was reached more frequently in the continuous vs. the intermittent feeding group (65.0% vs. 52.4%). However, a recent systematic review observed no differences in nutritional intake, mortality, or gastrointestinal intolerance between continuous and intermittent feeding [25]. During continuous feeding, the slow release of nutrients into the stomach is thought to reduce feeding tolerance, the risk of regurgitation, and respiratory complications. However, while intermittent feeding has been suggested to increase feeding intolerance (FI), gastric residual volume (GRV), and aspiration risk, there is no evidence showing that aspiration risk is higher in patients on intermittent feeding [26^▪▪^].

Moreover, intermittent feeding is considered more physiological as it mimics regular eating patterns, potentially maintaining regular gastrointestinal hormone secretion and digestion [27]. It may increase gut motility and enhance the release of postprandial gastrointestinal hormones and incretins involved in glucose control. However, no studies have compared strategies on gastric emptying and glucoregulatory hormone release in ICU patients. Studies that have assessed the effect of intermittent feeding on glycaemic variability show either increased glycaemic variability [28] or no differences in blood glucose levels [29]. These studies assessed 4–6 hourly glucose levels. Real-time continuous glucose monitoring may provide more insight into the glycaemic response and variability.

Noncontinuous feeding may attenuate muscle wasting due to increased plasma amino acid availability leading to increased muscle protein synthesis rates [28]. However, no studies have assessed the effect of bolus feeding on muscle metabolism in critically ill patients. Meal timing has been shown to play an essential role in metabolic health by preserving circadian rhythms in overweight, obese, or type 2 diabetes patients. With circadian rhythms being largely disrupted during critical illness, intermittent or cyclic feeding might effectively preserve circadian alignment [30]. Moreover, prolonged periods of fasting lead to improved glucose control, insulin sensitivity, improved lipid profiles, and the activation of ketogenesis and autophagy in healthy individuals. Nonetheless, this remains to be investigated in critically ill patients. Without evidence of the superiority of intermittent feeding, there is no reason to change our practice of continuous feeding in the ICU.

## FEEDING INTOLERANCE

Enteral FI is frequently encountered, especially in the early phase of ICU admission, potentially resulting in insufficient absorption of nutrients [31]. Nevertheless, a uniform definition of FI is lacking, which poses challenges for research [32]. A recent systematic review addressed various definitions used in studies to define FI. It is described as high GRV or other gastrointestinal (GI) symptoms; however, different cut-off points for volumes and combinations with clinical symptoms cause intra-study variety [33^▪▪^]. Furthermore, Blaser *et al.*[32] recommend that the FI-definition should comprise intake <80% of the target within the first 72 h of EN initiation and the presence of at least one GI-symptom while considering the optimization of non-EN-related factors (e.g., medication, GI-infection, and bowel anatomy).

FI has been shown to be associated with worse clinical outcomes, such as fewer ventilator-free days, extended ICU stay, and higher mortality rates [31,34]. However, numerous strategies to impact FI and improve nutrition delivery have been proposed. In a posthoc analysis of the TARGET-trial, patients with GRV >250 ml showed lower mortality rates when treated with prokinetics [34]. Conversely, in a meta-analysis, prokinetics did not lower mortality. However, reduced lengths of ICU and hospital stay were found [35^▪^]. Prebiotics, probiotics, or synbiotics do not significantly affect FI [36]. Moreover, energy-dense enteral feeds have resulted in a higher incidence of FI [34]. A recent meta-analysis showed that postpyloric feeding was associated with fewer GI complications, increased feeding adequacy, and reduced mechanical ventilation and ICU stay duration, although no difference in mortality was shown [37^▪^]. Thus, treatment with prokinetics and administering postpyloric feeding in patients not responding to prokinetics should be considered when FI emerges. A uniform definition of FI is urgently needed.

## NUTRITION IN CORONAVIRUS DISEASE 2019

The SARS-CoV-2 pandemic warranted research for specific nutritional strategies in the ICU. In approximately 56% of COVID-19 ICU patients, FI was present, and 52% of patients suffered from malnutrition. Among other factors, the hypermetabolic and prolonged catabolic state of COVID-19 patients make personalized and accurate nutrition treatment essential [38^▪▪^,^39^]. Furthermore, SARS-CoV-2 can attack the mucosal epithelium and cause gastrointestinal symptoms, increasing FI and malnutrition risk [40].

Early enteral nutrition, within 24–36 h of ICU admission or 12 h of intubation, showed to reduce mortality of COVID-19 ICU patients in a systematic review. However, no significant differences in the length of ICU and hospital stay or mechanical ventilation duration were observed [41^▪▪^]. Patients with an increased malnutrition risk, such as older and polymorbid patients, should be identified and treated accordingly. Supplementation of vitamins and trace elements, physical activity, oral nutritional supplements (ONS), and EN should be administered if necessary [42].

## POST-ICU NUTRITION

**FIGURE 1:**
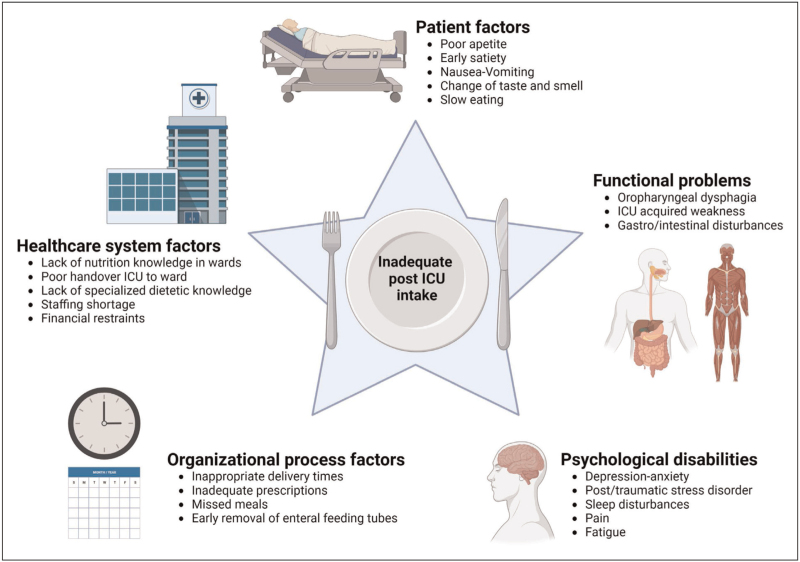
Overview of multifactorial causes contributing to inadequate post-ICU nutritional intake. Created with biorender.com. License ARH van Zanten: agreement number: UI24KC5DCW. ICU, intensive care unit.

No formal guidelines on nutrition therapy for post-ICU patients are available. However, energy and protein intake should likely be further increased during convalescence when inflammation resolves and elevated muscle protein breakdown rates decrease, particularly when combined with physical activity [43]. Several studies have shown poor feeding performance among post-ICU patients (50–70% of energy and protein adequacy) [1,44^▪^], highlighting the need for specific interventions. Inadequate intake in the post-ICU phase is multifactorial [44^▪^,^45^]. Several factors of poor feeding intake are summarized in Fig. 1.

In the PROSPECT-I study, Slingerland-Boot *et al.*[46^▪^] showed that removal of the nasogastric tube leads to an immediate decrease in daily energy (44.1%) and protein (50.7%) intake, suggesting that EN tapering protocols and introduction of ONS after tube removal are essential to optimize nutritional intake during ICU recovery. The causes of inadequate intake should be evaluated. Nutrient intake must be monitored, and continuity of nutritional treatment in the post-ICU phase in general wards and at home should be guaranteed.

## CONCLUSION

Nutrition therapy must be adjusted to the phases of the disease and convalescence. Significant scientific steps have been made toward achieving this goal. Early energy overfeeding should be avoided, although precise targets are lacking. Indirect calorimetry can guide energy targets after the initial phase. Individualized protein dosing warrants assessment of the LBM with BIA or ultrasound. There is more doubt about whether high protein intake improves clinical endpoints. However, it may mitigate muscle mass loss. Protein absorption in critical illness is normal, however, severe skeletal muscle anabolic resistance may limit the effects of high protein intake. Intermittent feeding cannot be recommended over continuous feeding. Feeding intolerance is common and can often be treated with prokinetics or postpyloric tubes. COVID-19 ICU patients are at risk for malnutrition and marked muscle loss. Early enteral nutrition may improve outcomes. Many patients’ post-ICU nutrition intake is poor, particularly when the feeding tube is removed early. Causes are multifactorial.

## Acknowledgements

*None.*

### Financial support and sponsorship

*None.*

### Conflicts of interest

*Professor Van Zanten reported receiving honoraria for advisory board meetings, lectures, research, and travel expenses from Abbott, AoP Pharma, Baxter, Cardinal Health, Danone-Nutricia, Dim-3, Fresenius Kabi, GE Healthcare, Medcaptain, Mermaid, Nestle-Novartis, Lyric, and Rousselot. The other authors have nothing to declare.*

## REFERENCES AND RECOMMENDED READING

Papers of particular interest, published within the annual period of review, have been highlighted as:

▪ of special interest
▪▪ of outstanding interest
